# Influence of Varying Airborne-Particle Abrasion Angles on Surface Properties and Flexural Strength of Feldspar Ceramic

**DOI:** 10.3390/jfb17080358

**Published:** 2026-07-26

**Authors:** Valerie Lankes, Andrea Coldea, John Meinen, Bogna Stawarczyk

**Affiliations:** 1 Department of Prosthetic Dentistry, LMU University Hospital, LMU Medizin, Ludwig-Maximilians-Universität München, Goethestraße 70, 80336 Munich, Germany; v.lankes@med.uni-muenchen.de (V.L.); andrea.coldea@med.uni-muenchen.de (A.C.); john.meinen@med.uni-muenchen.de (J.M.); 2 Biomaterials and Technology, Department Research, University Center for Dental Medicine Basel UZB, University of Basel, Mattenstrasse 40, CH-4058 Basel, Switzerland

**Keywords:** airborne-particle abrasion angle, alumina powder, feldspar ceramic, flexural strength, surface free energy, surface roughness

## Abstract

This study investigated the effects of airborne-particle abrasion angle and nozzle geometry on the surface free energy (SFE), surface roughness (Ra), and flexural strength (FS) of a feldspar ceramic. A total of 70 feldspar ceramic substrates were assigned to seven groups (*n* = 10). Six groups were air-abraded with 50 µm Al_2_O_3_ at 0.05 MPa for 10 s using a conventional nozzle at 20°, 45°, 70°, or 90°, or an experimental conical nozzle at 45° or 90°. The control group underwent hydrofluoric acid etching for 60 s. One-way ANOVA with partial eta-squared (η_p_^2^) was performed, followed by Scheffé post hoc tests. Treatment significantly affected SFE and Ra (*p* < 0.001). The 20° conventional-nozzle group showed the lowest SFE and Ra values (38.3 ± 11.3 mJ/m^2^ and 0.514 ± 0.033 µm, respectively). At 45°, the conventional nozzle produced higher SFE than the experimental nozzle, whereas at 90°, the experimental nozzle produced higher Ra. Mean FS ranged from 121 ± 8.5 to 131 ± 8.0 MPa, without significant differences among the groups (*p* = 0.074). Under the investigated low-pressure conditions, abrasion angle and nozzle geometry altered surface properties without a statistically detectable reduction in FS. Bond strength, aging durability, and clinical performance were not assessed. Therefore, the present findings alone do not justify a recommendation for replacing hydrofluoric-acid etching with airborne-particle abrasion.

## 1. Introduction

In dental practice and technology, airborne-particle abrasion is a fundamental process that plays a pivotal role in nearly all aspects of dental restoration production, preparation, and cementation/luting. The most used airborne-particle abrasion powder is alumina (Al_2_O_3_), which is available in various grain sizes ranging from 25 to 250 µm. The significance of airborne-particle abrasion extends across multiple facets of dental treatment and manufacturing [1]. It enhances the bond between dental restoration materials with cements or adhesives by roughening the surface and creating micromechanical retentive features that are crucial for the longevity and stability of fixed dental prostheses (FDPs) such as crowns, bridges, and veneers [2,3].

Airborne-particle abrasion is also routinely used in dental laboratories for divesting cast restorations after investment removal and for conditioning ceramic restorations surface before subsequent laboratory procedures or adhesive bonding [4,5]. Despite its numerous advantages in dentistry, airborne-particle abrasion can also cause damage to the surfaces of the treated restoration materials under certain conditions [6]. Such damage may compromise the integrity and functionality of restorations, thereby increasing the likelihood of failure [7,8,9]. Therefore, it is imperative to carefully select airborne-particle abrasion parameters to mitigate any adverse effects [10].

Well-studied parameters include the type of airborne-particle abrasion medium-such as alumina particles or glass beads, particle size, and pressure. High pressure can lead to microcracks and excessive surface abrasion depending on the restoration material; similarly, larger particle sizes can scratch or damage the surface, particularly with alumina. Feldspar ceramics with a high glassy phase are typically etched with hydrofluoric acid prior to bonding. Airborne-particle abrasion is avoided due to potential risks associated with strength reduction [11].

The main parameters of airborne-particle abrasion—abrasive medium, nominal pressure setting, and duration—can be adjusted by the operator; however, the actual jet energy at the ceramic surface varies considerably between devices and set-ups, even at the same nominal pressure. In addition, the outcome of airborne-particle abrasion is influenced by variables such as the angle of incidence, nozzle geometry, and working distance, although these factors have received considerably less scientific attention [12]. An oblique incidence of approximately 45° has frequently been used in experimental studies and has been proposed to reduce localized surface damage compared with perpendicular impact. However, the available evidence is limited and depends on the treated material and the remaining abrasion parameters [13,14,15,16,17]. At a 90° angle, the kinetic energy is concentrated on a smaller area, potentially increasing the risk of damage. Nevertheless, due to the complexity and variability of FDP geometries, achieving a consistent airborne-particle abrasion angle in practice is nearly impossible.

To the authors’ knowledge, the impact of different airborne-particle abrasion angles on the surface properties and mechanical strength of treated restoration material has not yet been investigated. Surface roughness and surface free energy are critical factors influencing the luting of restorations. A recent study indicates that silicate-based ceramic restorations achieve comparable bond strength values with adhesive luting after airborne-particle abrasion compared to those achieved by established but potentially hazardous hydrofluoric acid etching [18]. Silicate-based ceramics with lower flexural strength are particularly suitable for assessing subtle effects on material mechanical properties [19].

The null hypothesis posits that varying airborne-particle abrasion angles (20°, 45°, 70°, 90°) from two distinct nozzles (conventional and experimental) do not affect either the surface roughness (Ra) or free energy (SFE) or the biaxial flexural strength (FS) of a feldspar ceramic. Additionally, it is assumed that there will be no significant difference between etched and airborne-particle abraded feldspar ceramic.

## 2. Materials and Methods

In total, 70 feldspar ceramic substrates were prepared from CAD/CAM blocks (VITABLOCS Mark II, VITA Zahnfabrik, Bad Säckingen, Germany, LOT-No: 93910). The blocks were sectioned into specimens measuring 12 × 12 × 1.4 mm^3^ under water cooling, utilizing a rotational speed of 3700 rpm and a feed rate of 0.020 mm/s (Secotom-50, Struers, Ballerup, Denmark). Each specimen was then ground to a thickness of 1.2 mm (Abramin with 20 µm DiamondPad, Struers, Ballerup, Danmark) and subsequently polished (MD-Largo, Struers, Ballerup, Danmark) with diamond suspensions (9 and 3 μm, DiaPro Dac, Struers, Ballerup, Danmark) in accordance with DIN EN ISO 6872:2024 [20].

**Figure 1:**
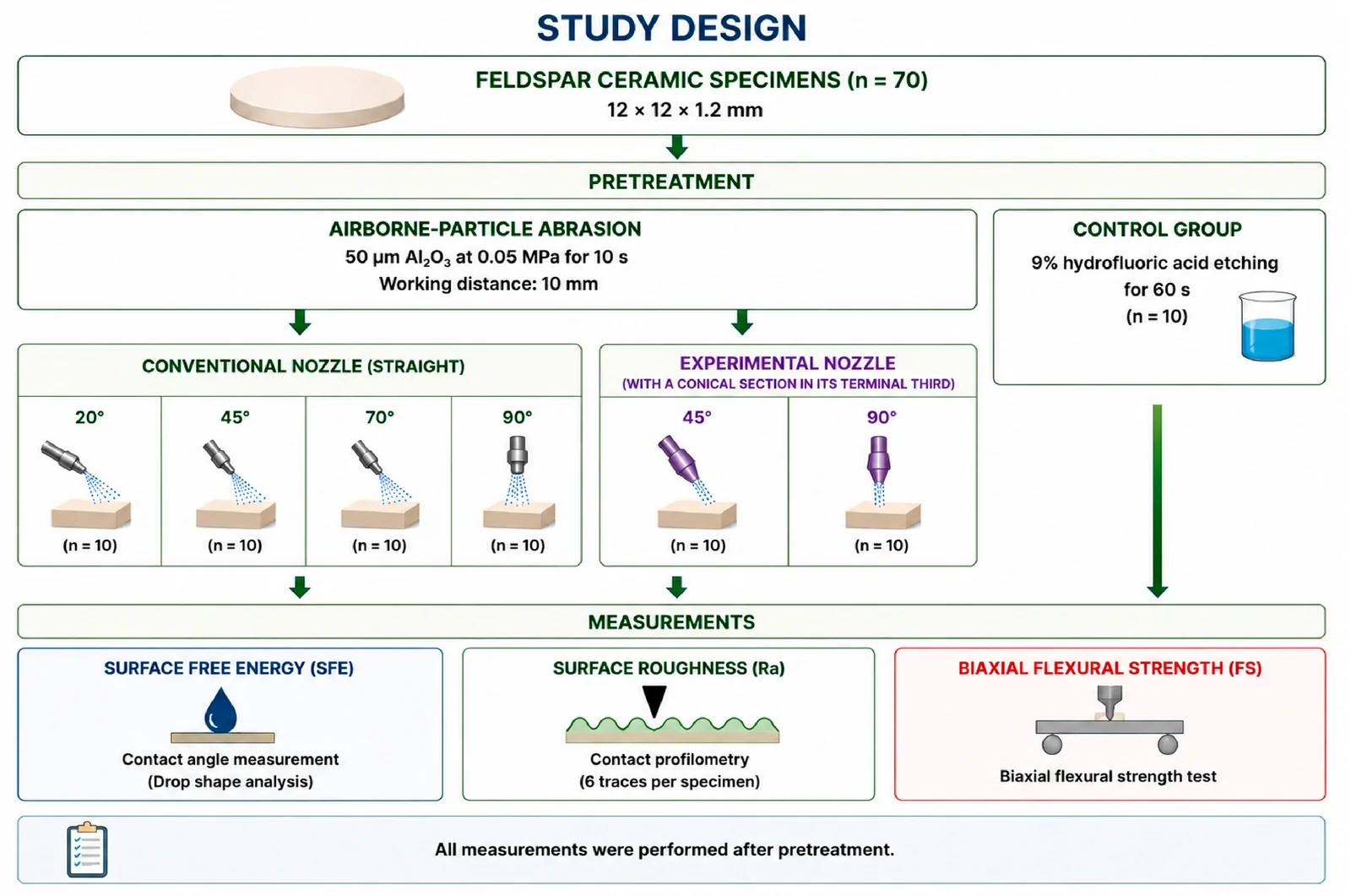
Overview of the experimental workflow. Seventy feldspar ceramic specimens were assigned to six airborne-particle abrasion groups using either a conventional straight nozzle (20°, 45°, 70°, and 90°) or an experimental nozzle with a conical terminal section (45° and 90°), and one hydrofluoric acid-etched control group. Surface free energy (SFE), surface roughness (Ra), and biaxial flexural strength (FS) were evaluated after surface pretreatment under standardized conditions.

The prepared substrates were divided into 7 groups (*n* = 10 per group), categorized by the angle of airborne-particle abrasion using a conventional straight nozzle (20°, 45°, 70°, 90°), an experimental nozzle with a conical section in its terminal third (45°, 90°), and a control group treated with 9% hydrofluoric acid etching for 60 s [21] (Ultradent Porcelain Etch 9%, Ultradent, Brunnthal, Germany, LOT BP6NG) (Figure 1).

**Figure 2:**
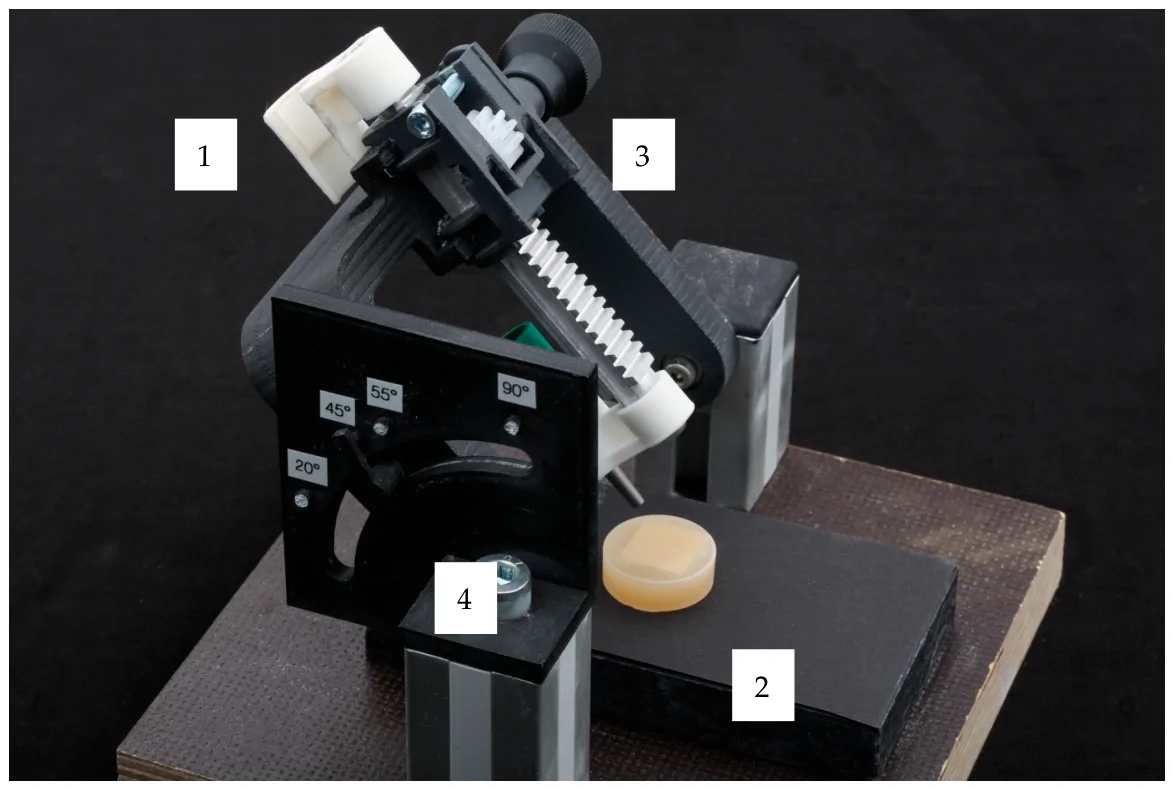
Custom-built airborne-particle abrasion device used to standardize the abrasion angle and the nozzle-to-specimen distance. The abrasion nozzle (1) was mounted on a movable carriage, and the feldspar ceramic specimen (2) was positioned on an adjustable specimen holder. The nozzle-to-specimen distance was adjusted using a rack-and-pinion mechanism (3). A pivoting angle-control plate with predefined angular positions (4) enabled reproducible adjustment of the nozzle axis between 20° and 90° relative to the specimen surface. The apparatus maintained the selected abrasion angle and working distance throughout the airborne-particle abrasion procedure.

Airborne-particle abrasion was performed using either a conventional straight nozzle or an experimental nozzle incorporating a modified conical terminal section. Both nozzles were based on a flow channel with an inlet diameter of 0.8 mm and were operated using the same airborne-particle abrasion device under identical operating conditions, including abrasive material, particle size, pressure, working distance, and application time. Airborne-particle abrasion was performed with 50 µm alumina powder (Cobra, Renfert, Hilzingen, Germany, LOT: 2548791) at a pressure of 0.05 MPa for 10 s. A fixed distance of 10 mm between the nozzle and substrate was maintained, and the investigated abrasion angles were adjusted and stabilized using a custom positioning device (Figure 2) integrated into a prototype device based on the Renfert Keramo 4, which was modified to enable more precise control of airborne-particle abrasion parameters, particularly pressure and abrasive powder flow rate. The experimental nozzle differed from the conventional straight nozzle exclusively in the geometry of its terminal section, whereas both nozzles shared the same flow channel inlet diameter of 0.8 mm and all remaining experimental parameters were kept constant. As the experimental nozzle is currently the subject of a pending patent application, detailed design specifications cannot be disclosed at this stage.

The substrates were cleaned in a 70% isopropanol solution (Otto Fischar, Saarbrücken, Germany) using an activated ultrasonic bath (Sonorex Digitec DC, Bandelin, Berlin, Germany) for 3 min. They were then carefully dried with compressed air before each substrate’s topography was analyzed using a digital microscope (VHX-970F, Keyence, Osaka, Japan) at 2000× magnification.

For SFE, a contact angle device (EasyDrop, Krüss, Hamburg, Germany) was utilized with both polar liquid (distilled water) and non-polar liquid (diiodomethane, CAS no. 75-11-6, Sigma-Aldrich, Steinheim, Germany). Contact angle measurements were performed within 30 min after airborne-particle abrasion under identical laboratory conditions (approximately 23 °C air-conditioned laboratory). For each specimen, three droplets of each measuring liquid were applied, and the mean contact angle was used for the calculation of the SFE. A drop with a volume of 1 µL was applied to the pretreated surface, and the drop shape was recorded (IEEE1394b–Stingray F-046, Allied Vision Technologies GmbH, Stadtroda, Germany) after 5 s. The contact angle of each water droplet was evaluated using the tangent 1 method, whereas that of diiodomethane was assessed using the circle method via DAS 25 software (EasyDrop, Krüss, Hamburg, Germany). The SFE values (mJ/m^2^) were calculated based on the Ström database. Relative humidity was not controlled during the measurements.

For Ra, a contact profilometer (Perthometer M2, Mahr, Göttingen, Germany) rec-orded 6 traces per specimen with each trace length set to 4 mm. After recording 3 traces in one direction horizontally, the specimens were rotated by 90° to record an additional 3 traces. The force exerted by the measuring needle was set to 0.00075 N.

FS was measured using a testing device equipped with 3 hardened steel balls (3.2 mm diameter) arranged at intervals of 120° on a supporting circle measuring 10.0 mm in diameter. The specimen was positioned centrally among the steel spheres and loaded with a flat punch having a diameter of 1.6 mm at a crosshead speed of 1 mm/min until fracture occurred. The biaxial flexural strength was calculated according to the formula [22]:

$$\sigma \max=f\left(\tau ,\nu \right)*\frac{F}{t^{2}}$$

$$f\left(\tau ,\nu \right)=0.323308+\frac{\left(1.30843+1.44301*\nu \right)*(1.78428-3.15347*\tau +6.679*\tau^{2}-4.62603*\tau^{3})}{1+1.71955*\tau }$$

with σ max: biaxial flexural strength [MPa]; F: fracture load [N]; t: specimen thickness [mm]; f: f-function; ν: Poisson’s ratio (0.18) [23]; and τ: the radius of the carrier disk [mm].

The assumption of normal distribution was tested using the Kolmogorov–Smirnov test. One-way ANOVA with partial eta-squared (η_p_^2^) followed by the Scheffé post hoc test was computed to analyze differences among groups. A two-group t-test investigated differences between the different nozzles within the same angle. All *p*-values below 0.05 were considered statistically significant. Statistical analyses were conducted using SPSS Statistics version V.29.0 (IBM, Armonk, NY, USA).

## 3. Results

No deviations from the normal distribution were observed, allowing for parametric analysis of all data (Table 1). Both SFE and Ra were influenced by the airborne-particle abrasion angle (η_p_^2^ = 0.588, *p* < 0.001) and the type of nozzle used (η_p_^2^ = 0.228, *p* < 0.001). However, no significant differences in FS values were observed between the airborne-particle abrasion groups and the control group (*p* = 0.074).

**Figure 3:**
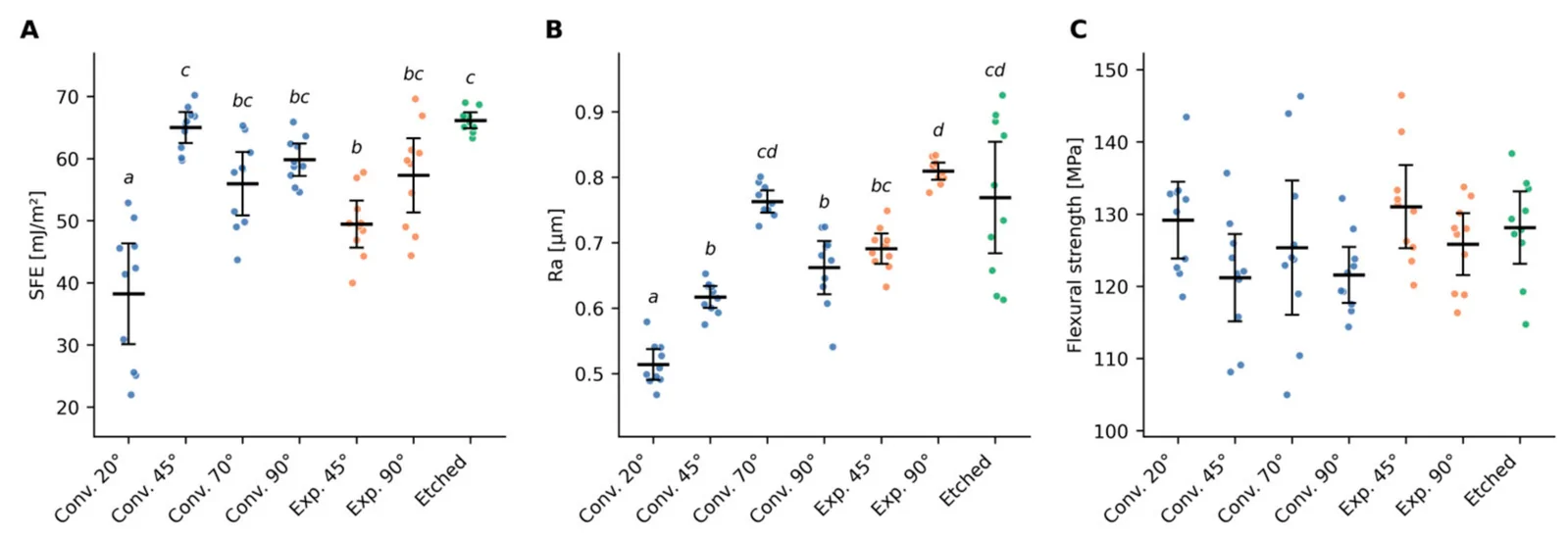
Individual measurements (*n* = 10 per group), group means (horizontal lines) and 95% confidence intervals (error bars) for (**A**) surface free energy, (**B**) surface roughness Ra, and (**C**) flexural strength. Groups not sharing a letter differ significantly (one-way ANOVA with Scheffé post hoc test, *p* < 0.05). Flexural strength showed no significant group differences.

An angle of 20° resulted in the lowest SFE (*p* < 0.038) and Ra (*p* < 0.001) of all groups (Figure 3). At 45°, the conventional nozzle produced a higher SFE than the experimental nozzle (*p* = 0.001); the experimental nozzle at 45° also remained below the SFE of the etched control (*p* < 0.001), while all other groups reached control-level SFE.

Regarding Ra, abrasion at 45° produced lower values than at 90° with the experi-mental nozzle (conventional: *p* < 0.001; experimental: *p* = 0.002). With the conventional nozzle, Ra at 45° and 90° remained below the etched control (*p* < 0.001 and *p* = 0.009), whereas the experimental nozzle at 45° did not differ from the control (*p* = 0.137). Abrasion at 70° produced higher Ra than 45° and 90° with the conventional nozzle (*p* < 0.001 and *p* = 0.017). At 90°, the conventional nozzle produced lower Ra values than the experimental nozzle (*p* < 0.001).

## 4. Discussion

The present study aimed to examine the effect of varying airborne-particle abrasion angles on both the surface characteristics, such as SFE and Ra, and on the FS of a feldspar ceramic. Contrary to expectation based on established parameters such as particle size, type, and pressure [24,25], the results indicate that while the angle of airborne-particle abrasion and the nozzle type significantly influence SFE and Ra, these factors do not compromise the FS of the ceramic material. Accordingly, the null hypothesis was only partially accepted, since significant differences were observed for the investigated surface properties, whereas the mechanical performance of the feldspar ceramic remained unaffected under the applied experimental conditions. Findings suggest that, at the investigated low abrasion pressure, the impact angle primarily modifies the surface condition of the ceramic rather than inducing structural defects that are sufficiently large to impair its flexural strength. These observations further support the concept that the outcome of airborne-particle abrasion depends on the interaction of multiple processing parameters rather than on individual variables alone. Besides particle size, pressure, and abrasive medium, the angle of incidence, nozzle geometry, and working distance should also be considered relevant determinants of the resulting surface characteristics. These parameters have received comparatively little scientific attention [12].

Although airborne-particle abrasion is routinely used for conditioning restorative materials prior to adhesive cementation, most previous investigations have focused on particle size, abrasive material, pressure, or application time rather than on the influence of the impact angle [2,3,24]. From a clinical perspective, however, the abrasion angle represents an important variable because a constant angle can rarely be maintained during the treatment of anatomically complex restorations, such as crowns, veneers, or fixed dental prostheses. Consequently, understanding whether deviations from the recommended application angle affect surface characteristics or compromise the mechanical integrity of feldspathic ceramics is of direct clinical relevance.

One important finding was that at a shallow angle of 20°, significantly lower SFE and Ra values were observed compared to other angles. This suggests that a more tangential application of the abrasive particles results in less pronounced texturing of the ceramic surface. This observation is mechanically plausible because the impact angle determines how the kinetic energy of the abrasive particles is distributed at the ceramic surface. At shallow incidence angles, a larger proportion of the particle momentum acts parallel to the surface, promoting sliding interactions rather than direct indentation. Consequently, abrasive particles are more likely to remove superficial asperities by tangential abrasion than to generate localized brittle fractures or pronounced material pull-outs within the glassy matrix. In contrast, increasing the impact angle increases the normal component of the impact force, thereby facilitating localized crack initiation and more effective surface roughening. Although the dental literature has scarcely investigated this mechanism directly, it is consistent with the surface modifications described after airborne-particle abrasion of dental ceramics [5,17]. One plausible explanation is that a flatter angle leads to a more glancing interaction between the particles and the ceramic surface, thereby removing or modifying fewer material grains and creating fewer pronounced topographic irregularities. Interestingly, reducing SFE and Ra may influence the wetting behavior of adhesives or cements, potentially affecting micromechanical retention and bonding performance [26]. However, the relationship between surface roughness, surface free energy, and adhesive performance is considerably more complex than often assumed. Surface roughness primarily contributes to micromechanical interlocking, whereas SFE influences the spreading and wetting behavior of adhesive monomers on the conditioned ceramic surface. The final bond strength additionally depends on chemical interactions between the ceramic surface and the silane coupling agent, the composition of the adhesive system, polymerization efficiency, and the stability of the adhesive interface during aging [3,7,16]. Therefore, neither SFE nor Ra alone should be regarded as surrogate parameters for durable adhesion [27].

However, although SFE and Ra are relevant parameters for adhesive bonding, they should not be interpreted as direct evidence of improved adhesive performance or long-term clinical success, since bond strength and aging behavior were not investigated in the present study. This distinction is particularly important because recent investigations have demonstrated that different airborne-particle abrasion protocols may achieve comparable immediate bond strength despite measurable differences in surface morphology [18,28]. Conversely, apparently favorable surface characteristics do not necessarily translate into improved long-term adhesion after thermal cycling or mechanical fatigue [16]. Consequently, the present findings should be interpreted as evidence that abrasion angle influences the physical surface characteristics of feldspathic ceramics rather than their adhesive performance. Future investigations should therefore combine surface analyses with bond strength testing and artificial aging protocols to determine whether the observed differences in SFE and Ra are clinically relevant.

Interestingly, the present findings indicate that surface characteristics appear to be considerably more sensitive to variations in airborne-particle abrasion angle than the mechanical integrity of the ceramic itself. While relatively small changes in the particle trajectory produced statistically significant differences in SFE and Ra, these alterations were not accompanied by a detectable reduction in flexural strength. This observation suggests that, under the investigated conditions, the induced surface modifications remained confined to the superficial layer and did not generate critical flaws capable of governing catastrophic fracture initiation during biaxial loading. This interpretation agrees well with previous investigations demonstrating that airborne-particle abrasion may substantially modify the surface morphology of feldspathic ceramics while maintaining their biaxial flexural strength [17,19]. Furthermore, the fracture behavior of brittle dental ceramics is governed primarily by the largest critical flaw within the stressed volume rather than by minor topographical changes confined to the outermost surface [22,23].

In contrast, treatment at angles closer to the frequently recommended 45° or the more perpendicular 90° angle tended to produce higher Ra and SFE values. Although a 45° angle has traditionally been suggested as a standard in the literature, our findings show that deviations from this angle lead to statistically significant changes in surface characteristics. This outcome emphasizes that even slight variations in treatment angle may alter the microtopography of the ceramic surface. From a clinical perspective, however, this observation should be interpreted with caution. During airborne-particle abrasion of anatomically complex restorations, such as veneers, inlays, onlays, or multi-unit fixed dental prostheses, maintaining a constant angle throughout the entire restoration surface is virtually impossible. Consequently, the present findings may better reflect the variability encountered in daily clinical practice than the idealized abrasion protocols commonly applied under laboratory conditions.

Nozzle geometry also emerged as a statistically significant factor. Compared with the experimental nozzle incorporating a conical section in its terminal third, the conventional straight nozzle produced higher SFE at 45° and lower Ra at 90°. One plausible explanation is that taper-induced plume divergence redistributes particle kinetic energy: at 45° it diminishes per-area impact, lowering surface activation, whereas at 90° it concentrates particles centrally, which increases roughness. Although this explanation remains speculative because particle trajectories were not directly analyzed, it agrees with fundamental principles of particle acceleration and flow dynamics, where nozzle geometry determines particle velocity distribution, impact density, and kinetic energy transfer to the target surface. Future studies employing computational fluid dynamics or high-speed particle imaging may help clarify these mechanisms.

Because the experimental nozzle was evaluated only at the shared angles of 45° and 90°, the present findings should not be interpreted as evidence of an overall superiority of either nozzle design. Rather, they demonstrate that nozzle geometry may influence surface characteristics under representative clinically relevant abrasion conditions. To the authors’ knowledge, the influence of nozzle design itself has rarely been investigated in dental biomaterials research, although it may represent an additional source of variability between different airborne-particle abrasion devices. Consequently, future studies comparing commercially available systems under standardized conditions are warranted.

Despite measurable differences in SFE and Ra, flexural strength values remained statistically comparable with those of the hydrofluoric acid (HF)-etched control across all angle–nozzle combinations, ranging from approximately 121 to 131 MPa. Although the experimental nozzle exhibited a numerically higher mean flexural strength at 45° than the conventional nozzle, this difference was not statistically significant and therefore should not be interpreted as an effect of nozzle geometry. Recent data demonstrate that feldspathic crowns air-abraded under higher pressure and a broad 35–90° angle window retained fracture loads comparable to HF-etched controls, even after 1.2 million chewing cycles [11]. Likewise, Hoffmann et al. [17] found that modifying Al_2_O_3_ particle size from 3 µm to 125 µm substantially shifted SFE and Ra yet left biaxial flexural strength statistically unchanged. The present findings therefore reinforce growing evidence that carefully controlled airborne-particle abrasion primarily modifies the superficial surface layer without necessarily introducing structural defects that reduce the fracture resistance of silica-based ceramics. This observation is consistent with fracture mechanical concepts proposed by Kelly and Denry, who described that the strength of brittle dental ceramics is governed primarily by the size and distribution of critical flaws rather than by moderate superficial roughening alone [24]. Similarly, Zhang and Lawn emphasized that clinically relevant damage is predominantly associated with deep subsurface crack formation rather than limited surface texture modifications [10].

These observations should not be interpreted as indicating that increasingly aggressive airborne-particle abrasion protocols are biologically or mechanically harmless. Previous investigations have shown that increasing pressure, particle size, or exposure time may substantially increase surface damage and may eventually compromise ceramic reliability [10,24,25]. Instead, the present study suggests that, within the investigated low-pressure protocol, varying the abrasion angle modifies the surface characteristics to a much greater extent than the mechanical behavior of the feldspathic ceramic.

Taken together with the present results, these studies corroborate that airborne-particle abrasion provides a mechanically safe yet surface-tunable alternative to hydrofluoric etching across a wide range of clinical parameters. Nevertheless, this conclusion should not be interpreted as evidence that airborne-particle abrasion can generally replace hydrofluoric acid etching for silica-based ceramics [29]. Hydrofluoric acid produces a fundamentally different etching pattern by selectively dissolving the glassy matrix, whereas airborne-particle abrasion predominantly generates mechanical surface alterations. Consequently, both pretreatment strategies rely on different adhesion mechanisms and should not be regarded as interchangeable solely on the basis of surface roughness or flexural strength measurements. Comprehensive evaluation of bond strength, failure modes, hydrolytic degradation, and long-term clinical performance remains necessary before recommendations regarding replacement of hydrofluoric acid can be made [30,31].

Lastly, these findings underscore the importance of ongoing research into parameter variability in airborne-particle abrasion. While many studies have focused on particle size, pressure, and medium type, the angle of application remains less explored. The present investigation therefore extends current knowledge by demonstrating that the impact angle represents an independent processing parameter capable of modifying the surface condition of feldspathic ceramics without measurably affecting their flexural strength under low-pressure conditions. These findings contribute to a more comprehensive understanding of airborne-particle abrasion and may support the future development of standardized pretreatment protocols for silica-based ceramic restorations.

A major limitation of this study is its use of relatively low pressure (0.05 MPa) for airborne-particle abrasion. Under clinical or laboratory conditions, it is quite possible that higher pressures are applied, which could alter both the surface properties and, potentially, mechanical properties of the restorative material differently [18]. However, the pressure level chosen in this study seemed appropriate given the comparatively low strength of the feldspar ceramic used. Higher abrasive pressures might have increased the risk of microcracks or more severe surface damage, thus distorting any influence attributed to abrasive angle.

Additional limitations should also be considered. Only one feldspathic CAD/CAM ceramic, one alumina particle size (50 µm), one application time, one nozzle distance, and one abrasive medium were investigated. Furthermore, surface characterization was limited to roughness and surface free energy, whereas high-resolution analyses such as scanning electron microscopy, three-dimensional optical profilometry, or fractographic evaluation could have provided additional insight into the mechanisms responsible for the observed surface modifications. Finally, no bond strength testing, artificial aging, cyclic fatigue, or clinical simulation was performed. Consequently, the present findings should be interpreted exclusively as material-related observations under standardized laboratory conditions and should not be extrapolated directly to clinical performance. Future investigations should evaluate different ceramic systems, broader pressure ranges, alternative particle sizes, long-term adhesive durability, and fatigue behavior to establish evidence-based recommendations for clinical airborne-particle abrasion protocols.

## 5. Conclusions

Within the limitations of this study, varying airborne-particle abrasion angles and nozzle geometry influenced the surface characteristics of feldspar ceramic. SFE and Ra varied depending on the applied treatment, whereas flexural strength remained unaffected under the investigated conditions. Airborne-particle abrasion performed at a pressure of 0.05 MPa resulted in flexural strength values comparable to those obtained after HF etching, while producing treatment-dependent differences in surface properties. At the shared abrasion angles, the conventional nozzle produced a higher SFE at 45°, whereas the experimental nozzle produced a higher Ra at 90°, indicating that nozzle geometry influences the resulting surface characteristics under otherwise identical airborne-particle abrasion conditions. Because the experimental nozzle was evaluated only at the shared angles of 45° and 90°, the present findings should not be interpreted as demonstrating a general advantage of either nozzle design. The present findings suggest that low-pressure airborne-particle abrasion may represent a promising alternative surface pretreatment for feldspar ceramics. However, the observed differences in Ra and SFE should not be interpreted as direct evidence of improved adhesive performance, since bond strength, aging durability, failure modes, and clinical performance were not investigated. Furthermore, these results are specific to the investigated airborne-particle abrasion protocol (0.05 MPa) and to the tested feldspar ceramic, 50 µm alumina particles, abrasion duration, nozzle distance, and airborne-particle abrasion devices and should not be generalized to other airborne-particle abrasion protocols or higher abrasion pressures, which may result in different surface characteristics and mechanical behavior. Further studies are required to evaluate the influence of different pressure settings and to assess the long-term clinical performance of this surface treatment approach.

## Figures and Tables

**Table 1:** Descriptive statistics: Mean ± Standard Deviation and 95% confidence intervals (CI) of measured SFE [mJ/m^2^], Ra [µm] and FS [MPa] per air-abrasion nozzle, angle, and etched groups.

	SFE [mJ/m^2^]		Ra [µm]		FS [MPa]	
	Mean ± SD	95% CI	Mean ± SD	95% CI	Mean ± SD	95% CI
**Conventional nozzle**						
20°	38.3 ± 11.3	(30; 47)	0.514 ± 0.033	(0.490; 0.537)	129 ± 7.44	(122; 135)
45°	65.0 ± 3.5	(62; 68)	0.617 ± 0.023	(0.600; 0.634)	121 ± 8.45	(114; 128)
70°	56.0 ± 7.2	(50; 62)	0.763 ± 0.024	(0.746; 0.780)	125 ± 13.01	(114; 135)
90°	59.8 ± 3.6	(57; 63)	0.662 ± 0.057	(0.621; 0.703)	122 ± 5.40	(116; 126)
**Experimental nozzle**						
45°	49.5 ± 5.3	(45; 54)	0.691 ± 0.032	(0.668; 0.714)	131 ± 8.04	(124; 137)
90°	57.3 ± 8.3	(51; 64)	0.809 ± 0.018	(0.796; 0.822)	126 ± 6.01	(120; 131)
**Etched**	66.2 ± 1.8	(64; 68)	0.768 ± 0.119	(0.684; 0.854)	128 ± 7.01	(123; 134)

## Data Availability

The original contributions presented in the study are included in the article, further inquiries can be directed to the corresponding author.
